# The Medical Critic and Psychological Journal

**Published:** 1861-05

**Authors:** 


ART. XII.—*The Medical Critic and Psychological Journal.* Edited by FORBES WINSLOW, M.D., etc. No. 1, Jan., 1861. Churchill, London.

DR. WINSLOW'S *Journal of Psychological Medicine and Mental Pathology* has always been numbered among our most valued exchanges, every number being replete with interesting and highly useful matter. "In forwarding No. 1 of *The Medical Critic,* the first of a new series of Dr. Winslow's *Psychological Journal,* the publisher respectfully directs the attention of the editor to the fact that it is to be no longer exclusively psychological in its character, but that subjects of general interest to the medical profession, as well as to the public, are to be discussed in its pages." Although the journal is to be enlarged to two hundred pages, we regret to see that a series of essays, similar in literary character to the leading articles of *The Times,* and papers in the *Saturday Review,* are to be introduced. In its former guise, the periodical was so excellent that it could scarcely be improved upon; and we are, therefore, of the opinion that the new feature forms a very questionable addition to its pages.

